# Effects of Cocoa-Rich Chocolate on Blood Pressure, Cardiovascular Risk Factors, and Arterial Stiffness in Postmenopausal Women: A Randomized Clinical Trial

**DOI:** 10.3390/nu12061758

**Published:** 2020-06-12

**Authors:** Irene A. Garcia-Yu, Luis Garcia-Ortiz, Manuel A. Gomez-Marcos, Emiliano Rodriguez-Sanchez, Cristina Agudo-Conde, Jesus Gonzalez-Sanchez, Jose A. Maderuelo-Fernandez, Jose I. Recio-Rodriguez

**Affiliations:** 1 Instituto de Investigación Biomédica de Salamanca (IBSAL), Unidad de Investigación de Atención Primaria de Salamanca (APISAL), Servicio de Salud de Castilla y León (SACyL), 37005 Salamanca, Spain; lgarciao@usal.es (L.G.-O.); magomez@usal.es (M.A.G.-M.); emiliano@usal.es (E.R.-S.); cagudoconde@yahoo.es (C.A.-C.); jesusgonzsan@usal.es (J.G.-S.); jmaderuelo@saludcastillayleon.es (J.A.M.-F.); donrecio@usal.es (J.I.R.-R.); 2 Departamento de Ciencias Biomédicas y del Diagnóstico, Universidad de Salamanca, 37007 Salamanca, Spain; 3 Departamento de Medicina, Universidad de Salamanca, 37007 Salamanca, Spain; 4 Departamento de Enfermería y Fisioterapia, Universidad de Salamanca, 37007 Salamanca, Spain

**Keywords:** arterial pressure, vascular stiffness, risk factors, chocolate, postmenopause

## Abstract

This study aimed to evaluate the effects of the intake of 10 g of cocoa-rich chocolate on blood pressure, other cardiovascular risk factors, and vascular structure and function in postmenopausal women. A total of 140 postmenopausal women participated in this randomized and controlled parallel clinical trial. For six months, the intervention group (IG; *n* = 73) consumed daily 10 g of chocolate (99% cocoa) added to their usual food intake, whereas the control group (CG; *n* = 67) did not receive any intervention. Blood pressure, pulse pressure (PP), cardio-ankle vascular index (CAVI), ankle-brachial index (ABI), brachial-ankle pulse wave velocity (baPWV), augmentation index, and laboratory variables were measured at baseline and six months. ANCOVA analyses adjusted for baseline values revealed no significant differences for systolic blood pressure (−1.45 mm Hg; 95% confidence interval (CI): −4.79, 1.88; *p* = 0.391) or baPWV (0.18 m/s; 95% CI: −0.14, 0.50; *p* = 0.263) between groups. A decrease in PP was observed in the IG compared to the CG (−2.05 mm Hg; 95% CI: −4.08, −0.02; *p* = 0.048). The rest of the vascular structure and function parameters and other measured variables remained unchanged. The daily intake of 10 g of cocoa-rich chocolate seems to provide little improvement to cardiovascular health, but neither does it cause any adverse effects on the parameters evaluated in postmenopausal women in the long term.

## 1. Introduction

The risk of cardiovascular disease is lower in women than in men [1]. However, with the beginning of menopause, there is an increased risk associated with the estrogen deficiency that takes place in that period [2].

Interventions aimed at the prevention of cardiovascular disease are especially important in populations with increased risk. In the last few years, non-pharmacological treatments and other prevention strategies are being studied, such as dietary counseling, with the aim of improving the health of people with high risk of suffering from cardiovascular disease [3].

In this regard, flavonoids, which are a group of polyphenols, have been widely studied and their consumption has been related to beneficial effects on health. These benefits include reducing the risk of cerebrovascular disease, lung cancer, type 2 diabetes, or asthma incidence [4] and the risk of colon and rectal cancer [5], as well as preventing ischemic heart disease [6], among others. The beneficial effects of flavonoids have been attributed to their antioxidant properties [7]. Although recent studies provide further explanation of flavonoids’ mechanism of action related to reactive oxygen species (ROS) scavenging, immune modulation, cell cycle regulation, epigenetic modification, and genetic regulation of metabolism [8,9].

Different reviews suggest that a polyphenol-rich diet can have protective effects on cardiovascular health [10,11]. Lockyer et al. reported that phenolic-rich olive leaf extract intake decreased blood pressure as well as lipid profile in pre-hypertensive males [12]. In addition, Fuchs et al. concluded that single doses of tea theaflavins and catechins had moderate effects on peripheral microcirculation in healthy subjects [13], while other authors found that a supplementation with green tea catechin extract reduced total cholesterol (TC) and low-density lipoprotein (LDL) cholesterol levels in postmenopausal women [14]. Other studies have evaluated the effects of intaking chocolate and cocoa with a high concentration of polyphenols. The results of a systematic review showed that these products improved flow-mediated dilatation and reduced insulin resistance, having acute and chronic beneficial effects on cardiovascular health [15].

The available evidence suggests that cocoa-rich chocolate can have beneficial effects on arterial stiffness, vascular function, and cardiovascular risk factors in postmenopausal women. In a trial conducted in this population, which evaluated the daily and alternate-day intake of 17 g of flavonoid-rich cocoa, an improvement in arterial stiffness was observed through a decrease in pulse wave velocity (PWV) [16]. Moreover, arterial pressure and pulse pressure (PP) decreased after intervention, as well as some cardiovascular risk factors, such as glucose and triglycerides, with no other changes in the lipid profile. The results of another study showed a decrease in cerebral artery blood flow velocity in postmenopausal women after the intake of dark chocolate (80% cocoa) and milk chocolate (35% cocoa) [17].

However, there are very few clinical trials in the literature focused on evaluating these effects using a commercial compound that reproduces normal clinical conditions. Previous studies have a small sample size and, in addition, a short follow-up time [16,18,19].

The aim of this study was to evaluate the effects of the intake of 10 g of cocoa-rich chocolate (99%) on blood pressure, other cardiovascular risk factors, and vascular structure and function in postmenopausal women.

## 2. Materials and Methods

### 2.1. Design and Setting

The design corresponds to a randomized and controlled clinical trial with two separate groups. The study was carried out in the Primary Care Research Service of Salamanca (APISAL) (Salamanca, Spain), which is part of the Biomedical Research Institute of Salamanca (IBSAL) and the Spanish Research Network for Preventive Activities and Health Promotion in Primary Care (redIAPP). The study was conducted between June 2018 and August 2019. This clinical trial was registered at clinicaltrials.gov provided by the US National Library of Medicine as NCT03492983. The results reported in this manuscript are primary outcomes of the study.

### 2.2. Study Participants and Recruitment

**Figure 1:**
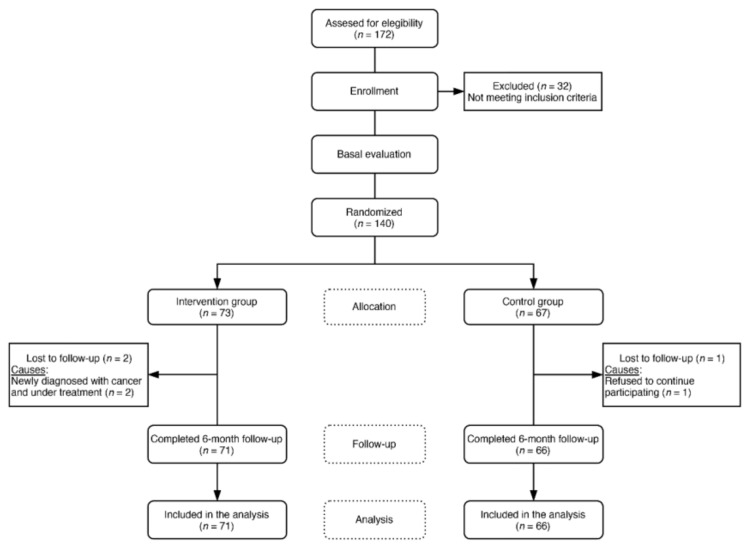
Flow chart of postmenopausal women through the study.

A consecutive sampling was carried out in the doctor’s offices of four urban primary care centers in Salamanca (Spain). Women who met the selection criteria and signed the informed consent for participation were recruited. A total of 140 women aged 50–64 years and in postmenopausal period, defined by amenorrhea for at least 12 consecutive months, were included in the trial. Thirty-two women were not included due to one or more of the exclusion criteria. Exclusion criteria were personal history of cardiovascular disease, personal background of diabetes mellitus, arterial hypertension, or dyslipidemia in pharmacological treatment, hypocaloric diets, clinically demonstrable neurological and/or neuropsychological disease, and treatment with hormone replacement therapy. Women with a usual consumption of more than 210 g of cocoa per week (g/wk) and intolerance and/or allergy to cocoa or any of the components of the supplement were also excluded (Figure 1).

### 2.3. Sample Size

The size of the sample was estimated based on the potential modification of the main variable, i.e., systolic blood pressure (SBP). Considering given alpha and beta risks of 0.05 and 0.20, respectively, in bilateral contrast and a standard deviation (SD) of 5.8 mm Hg, 140 participants (70 per group) were necessary to detect a minimum difference of 2.9 mm Hg in SBP between the two groups. A predicted drop-out rate of 10% during follow-up was taken into account. This estimate considered the results obtained in a similar study in which a decrease in SBP of 6.5 ± 5.8 mm Hg was observed [19].

### 2.4. Procedures and Randomization

All participants made a baseline visit and a visit at 6 months after the first visit, in which the study variables were measured (Figure 1). The intervention group (IG) made 5 additional resupply visits at 1, 2, 3, 4, and 5 months from the first visit, during which no other procedure was carried out apart from providing them with the necessary chocolate until the next visit and the collection of a calendar with a record of the chocolate intakes performed.

The participants were randomly distributed into two groups, namely the intervention group (*n* = 73) and the control group (*n* = 67). The assignation sequence was generated by an independent researcher using Epidat V.4.2 software [20]. The participants received their randomization number based on the order of their baseline evaluation visit and remained hidden until they were assigned to each group. To ensure that the blinding was maintained, the patients were given clear instructions not to disclose which treatment they had been randomized to while being interviewed by the blind assessors. Information on treatment allocation was stored in a secure locker in case of emergency unblinding.

Due to the characteristics of the intervention, it was not possible to blind all participants. To minimize cross-contamination between groups, the researcher who conducted the evaluations was different from the researcher who carried out the resupply of chocolate for the IG.

### 2.5. Intervention

The control group (CG) participants did not receive any type of intervention. The IG participants were given chocolate with a cocoa concentration of 99% and the instructions for daily consumption of 10 g of this compound added to their usual food intake. After the basal evaluation, they were given consumption and conservation instructions, recommending the daily intake of chocolate to be at the same time each day. In addition, they were given a calendar to record the time and the intake of each day, which was given back to the researchers in each resupply visit.

The daily nutritional contribution of 10 g of this chocolate, as stated by the manufacturer, is 59 kcal, 0.8 g of carbohydrates, 1.5 g of proteins, and 5.1 g of fat, of which 3.1 g are saturated fat. The determination and quantification of individual phenolic compounds (mg/g) in the cocoa were carried out by high-performance liquid chromatography coupled to a photodiode array detector and mass spectrometer (HPLC–DAD–ESI/MS). The composition in polyphenols was quantified from the areas of their chromatographic peaks by comparison with calibration curves prepared with the external standards of each compound. The compounds procyanidin trimer and procyanidin A were quantified by the procyanidin dimer B2 calibration curve, due to the lack of standards neither commercial nor isolated in the laboratory. The contribution of polyphenols in 10 g of this product is 65.4 mg. The polyphenolic profile of this compound is shown in Table 1. The participants of both groups were requested to continue with the dietary pattern they usually followed without modifying their eating habits during the study period.

### 2.6. Main Outcomes

#### 2.6.1. Blood Pressure Measurements

SBP, diastolic blood pressure (DBP), and heart rate (HR) were measured using a validated Omron M10-IT sphygmomanometer (Omron Healthcare, Kyoto, Japan). Three measurements were made following the recommendations of the European Society of Hypertension [21]. Then, PP was calculated as the difference between SBP and DBP, whereas the mean blood pressure was estimated using the following formula: DBP + 1/3 (SBP-DBP).

#### 2.6.2. Evaluation of Vascular Structure and Function

The cardio-ankle vascular index (CAVI), ankle-brachial index (ABI), and brachial-ankle pulse wave velocity (baPWV) were evaluated using a Vasera VS-2000 (Fukuda Denshi, Tokyo, Japan) device, following the manufacturer’s instructions.

The CAVI and the baPWV values were calculated using the equation as published by Shirai et al. [22]. The ABI was calculated by dividing the higher of the two ankle systolic pressures by the highest measurement of the two systolic pressures in the arm [23].

Vascular function was evaluated through central augmentation index (cAIx), central augmentation index corrected for a heart rate of 75 bpm (cAIx75), and peripheral augmentation index (pAIx). These measurements were carried out using a wrist-worn device, developed by Microsoft Research (Redmond, WA, USA), which includes an applanation tonometer placed over the radial artery [24]. The CAIx was normalized to a standard HR of 75 bpm using the equation published by the manufacturers of the Sphygmocor device (AtCor Medical Pty Ltd, West Ryde, Australia) [25]. The PAIx was calculated following the equation proposed by Munir et al. [26].

#### 2.6.3. Laboratory Variable Assessment

The following laboratory variables were measured: plasma fasting glucose values (mg/dL), plasma lipid profile (TC (mg/dL), total triglycerides (mg/dL), high-density lipoprotein (HDL) cholesterol (mg/dL), LDL cholesterol (mg/dL)), as well as creatinine (mg/L) and serum insulin (mg/dL) concentrations. Insulin resistance was determined using the Homeostasis Model Assessment Insulin Resistance index (HOMA-IR), which was estimated using the following equation: Fasting glucose (mmol/L) × insulin (mU/mL)/22.5. These laboratory variables were evaluated after blood extraction in at least 10–12 h of fasting, between 08:00 a.m. and 10:00 a.m. The participants were requested to avoid the consumption of chocolate and other polyphenol-rich products 24 h prior to the blood extraction.

#### 2.6.4. Other Measurements

1. Body Weight and Body Mass Index

Body weight was measured twice with an electronic scale (Scale 7830, Soehnle Professional, Backnang, Germany) after proper calibration (accuracy ± 0.1 kg). Height was measured by recording the average of two readings rounded to the nearest centimeter using a stadiometer (Seca 222, Medical Scale and Measurement System, Birmingham, UK). Both measurements were made with the subject barefoot and wearing light clothing. Body mass index (BMI) was calculated by dividing weight (kg) by height squared (m^2^). Based on baseline BMI cutoff value, subjects were classified into subgroups of overweight (BMI 25–29.9 kg/m^2^) and obese condition (BMI ≥ 30 kg/m^2^) [27].

2. Clinical and Sociodemographic Variables

At the baseline visit, information on clinical and sociodemographic variables was collected via questions about age, marital status, and educational level. The personal history of gestational diabetes, untreated hypertension and dyslipidemia, and the prescribed pharmacological treatment were recorded, as well as the time before diagnosis of menopause.

3. Adherence to the Intervention

Adherence was calculated as the percentage of days of chocolate intake with respect to the theoretical total days, according to the data recorded in the calendars of each IG participant.

4. Evaluation of Chocolate Consumption and Habitual Diet

Chocolate consumption was assessed at each evaluation visit by a series of questions about the amount, type, and frequency of consumption in the period between visits.

The nutritional composition of the habitual diet, which includes the distribution of macronutrients and energy consumption, was evaluated with a reminder of 24 h recorded for 3 non-consecutive days, prior to each evaluation day. These data were recorded and processed using the EVIDENT app [28].

The methods used for measuring other variables, such as physical activity, alcohol consumption, and smoking habits, are described in the previously published study protocol [29].

### 2.7. Data Collection Procedure, Data Management, and Monitoring

The collection of data in each evaluation visit was conducted by a nurse who had been previously trained for the task. Each participant was identified through a unique code that identified the data gathered in each of the measurements. With this, a database was created, which could only be accessed by the researchers of the study. The principal investigator carried out a data cleaning and clearing process in the database at the end of the study.

### 2.8. Ethical Considerations

The study was approved by the Clinical Research Ethics Committee of the Salamanca Health Area (CREC of the Health Area of Salamanca) in February 2018 (ethic approval code: PI11812/2017). The participants provided a signed informed consent, in accordance with the Declaration of Helsinki. The subjects were informed of the objectives of the project as well as the risks and benefits of the explorations to be carried out. The confidentiality of the participants’ data was guaranteed at all times, in accordance with the provisions of the Organic Law 3/2018, December 5th, of Personal Data Protection and guarantee of digital rights, and Regulation (EU) 2016/679 of the European Parliament and of the 27 April 2016 Council of Data Protection (RGDP), and under the conditions established by the national law 14/2007 of biomedical research.

### 2.9. Statistical Analyses

The statistical analysis was carried out following the study protocol [29]. The data were verified for normal distribution and most data were considered normally distributed. The characteristics of the study population are presented as mean and standard deviation for the continuous variables and as distribution of frequencies for the qualitative variables. To evaluate the comparability in the baseline evaluation between the two study groups, the chi-squares test was used for qualitative variables and the Student’s *t*-test was used to compare the means between the two groups.

The effects of chocolate consumption on the outcome measures (blood pressure, arterial stiffness, and cardiovascular risk factors), were evaluated using the Student’s *t*-test to compare the means between the two groups. To analyze the changes at 6 months from baseline in the outcome measures within the same group, the Student’s *t*-test for paired data was used. Analysis of covariance (ANCOVA) was performed to compare the effects, using the pre-values as covariates of the corresponding post-values. Intergroup differences are presented as means and 95% confidence interval (CI).

Subgroup analyses were conducted considering the presence or absence of overweight or obesity as baseline condition to evaluate SBP, DBP, and PP, using the Student’s *t*-test. These variables were also analyzed based on the way in which the chocolate was consumed (plain, with coffee/tea, with other foods, etc.) through ANOVA, and a post hoc test was conducted when a significant difference was found.

All analyses were performed using SPSS V.23.0 (IBM Corp., Armonk, NY, USA), establishing an alpha risk of 0.05 as the limit of statistical significance.

## 3. Results

### 3.1. Baseline Characteristics of the Study Groups

Of the 140 women included in the study (73 in the IG and 67 in the CG), there were two losses in the follow-up of the IG due to newly diagnosed cancer that required treatment and one loss in the CG who had refused to continue participating. Therefore, 137 women completed the study and were included in the analysis, with 71 in the IG and 66 in the CG (Figure 1).

Table 2 shows the baseline characteristics of the participants. As can be seen, no differences were observed between the two groups. The mean age in the IG and CG was 57.1 ± 3.5 and 57.5 ± 3.8 y, respectively. At the time of the baseline evaluation, the time lapsed from the beginning of menopause was similar in IG and CG. The consumption of chocolate as well as the consumption of chocolate with over 70% cocoa were also similar in both groups.

The mean adherence to the intervention was 97.6% ± 3.34%, with a minimum of 80.6% and a maximum of 100%.

### 3.2. Cardiovascular Risk Factors and Blood Pressure

The results of the effects on cardiovascular risk factors and blood pressure adjusted for baseline values are shown in Table 3. No significant differences were observed between groups for SBP (*p* = 0.391) or DBP (*p* = 0.622). There was a decrease in PP in the IG in contrast to the increase observed for this parameter in the CG, after adjustment for pre-values (*p* = 0.048). The levels of TC (*p* = 0.758), LDL cholesterol (*p* = 0.556), or HDL cholesterol (*p* = 0.795) did not significantly differ between groups. Likewise, there were no differences in body weight between the study groups or in the levels of glucose, insulin, or HOMA-IR.

Considering the way in which the chocolate was consumed (plain, with coffee/tea, with other foods, etc.), no significant differences were found for PP in the CG (0.55 mm Hg; 95% CI: −1.19, 2.30) compared to the participants who consumed the chocolate with coffee or tea (−1.75 mm Hg; 95% CI: −5.27, 1.77) and to those who consumed it without mixing it with other foods or liquids (−1.92 mm Hg; 95% CI: −4.35, 0.52) (*p* = 0.329).

**Figure 2:**
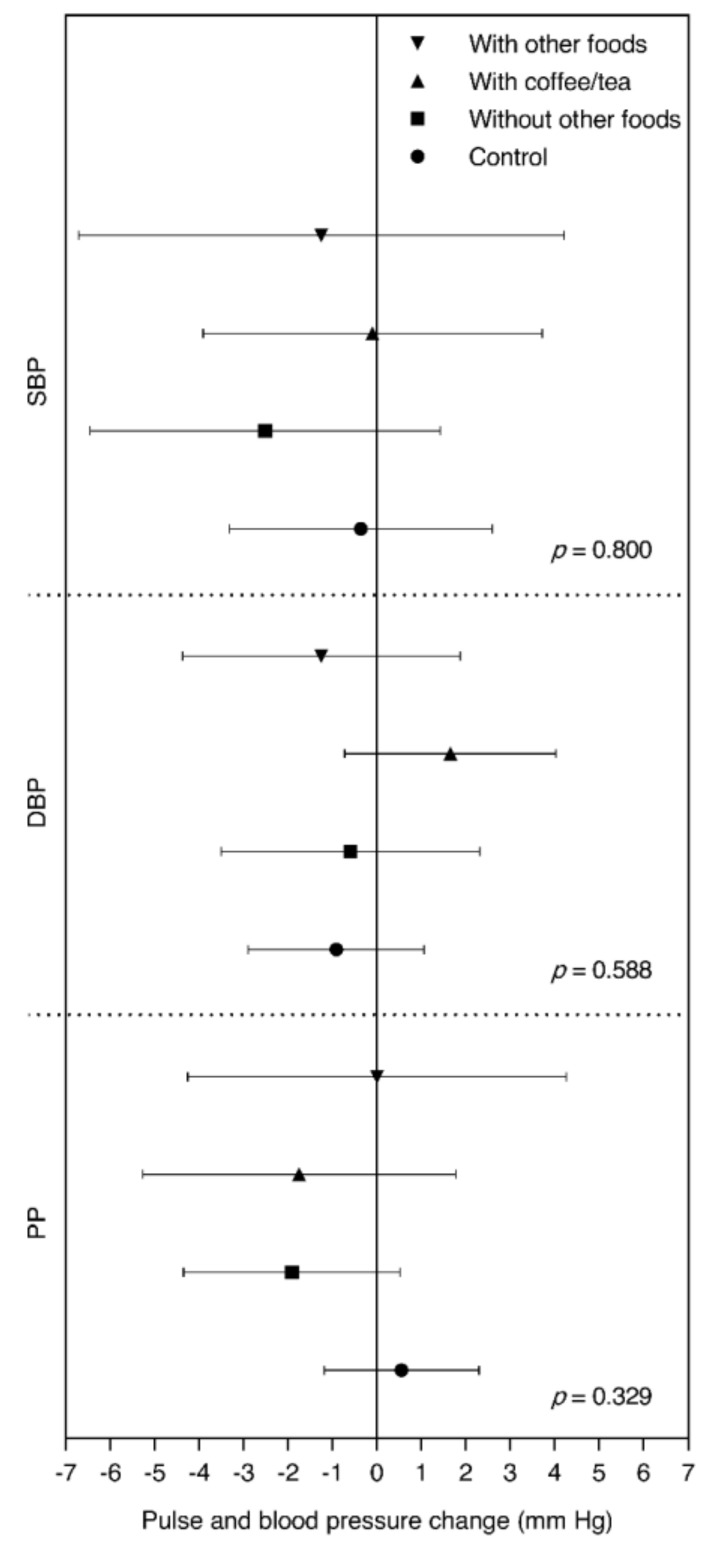
Changes in diastolic blood pressure (DBP), systolic blood pressure (SBP), and pulse pressure (PP) by the mode of chocolate intake in postmenopausal women participants. Values are differences in means (95% CI). *n* = 14 in subgroup that consumed chocolate with other foods, *n* = 20 in subgroup that consumed chocolate with coffee/tea, *n* = 36 in subgroup that consumed chocolate without other foods, *n* = 66 in control group. *p*-values from ANOVA for differences in DBP, SBP, and PP between all subgroups are shown.

Moreover, in this subgroup categorization, no changes were observed on SBP in the subgroup of participants who had chocolate on its own (−2.51 mm Hg; 95% CI: −6.45, 1.42) and in those who had it with other foods (−1.25 mm Hg; 95% CI: −6.71, 4.21) (*p* = 0.800). No statistically significant differences were found between groups in any case (Figure 2).

In the subgroup analysis based on the presence or absence or overweight or obesity as baseline condition, a sharp decrease of SBP was observed in the subjects of the IG with overweight or obesity (−4.64 mm Hg; 95% CI: −8.32, −0.96), in contrast to the increase observed in the CG (1.13 mm Hg; 95% CI: −2.15, 4.41) (*p* = 0.020).

**Figure 3:**
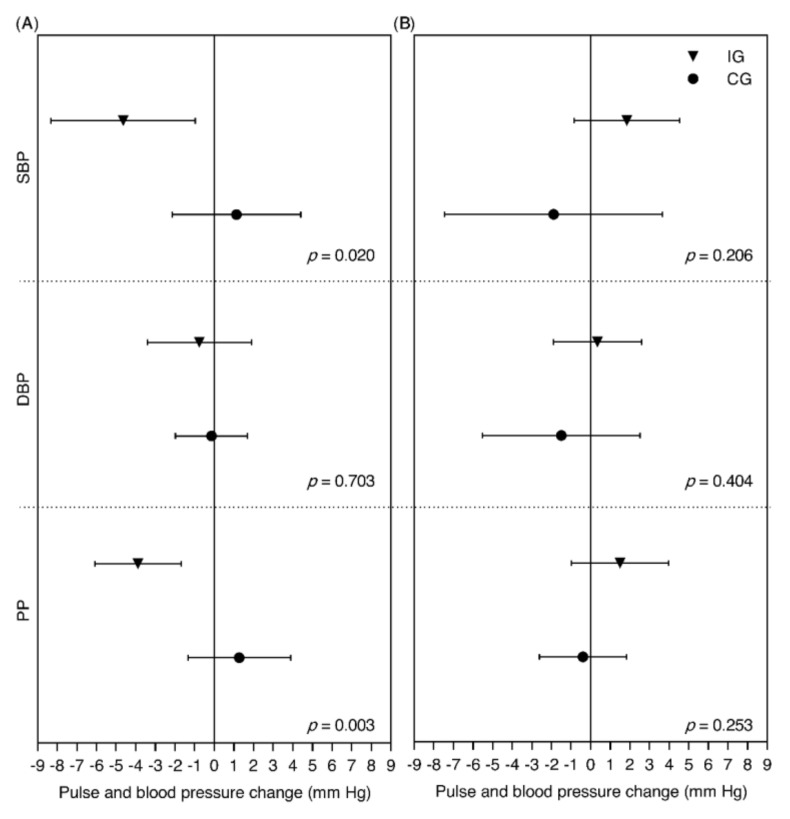
Subanalysis of changes in diastolic blood pressure (DBP), systolic blood pressure (SBP), and pulse pressure (PP) by overweight or obesity as a baseline condition in postmenopausal women participants. (**A**) Subgroup with overweight/obesity (intervention group (IG): *n* = 39; control group (CG): *n* = 38). (**B**) Subgroup without overweight/obesity (IG: *n* = 31; CG: *n* = 28). Values are differences in means (95% CI). *p*-values from Student’s *t*-test for differences in DBP, SBP, and PP between IG and CG are shown.

Similarly, PP decreased in the subjects of the IG with overweight or obesity (−3.88 mm Hg; 95% CI: −6.09, −1.68), in contrast to the increase in the subjects of the CG with overweight or obesity (1.28 mm Hg; 95% CI: −1.34, 3.89) (*p* = 0.003) (Figure 3).

### 3.3. Arterial Stiffness Parameters and Vascular Function

After adjusting for baseline values, no intergroup differences were found for any of the arterial stiffness parameters or vascular function (Table 4).

## 4. Discussion

### 4.1. Main Findings

The results did not show differences in SBP or DBP between groups, although a decrease in PP was observed in the IG compared to the CG. The levels of TC, LDL cholesterol, and HDL cholesterol, as well as the body weight, were similar in the two groups. Likewise, no differences were found in the levels of glucose, insulin, or HOMA-IR. No relevant changes were observed regarding the evaluation variables of vascular structure and function.

### 4.2. Discussion of Blood Pressure Results

Okamoto et al. [16], who administered a cocoa compound daily or in alternate days to postmenopausal women randomized in two groups, observed that values of SBP, DBP, mean arterial pressure (MAP), and PP decreased significantly with respect to the control group. A PP decrease associated with a greater consumption of chocolate has also been observed in studies with healthy adults [30]. Moreover, in a meta-analysis of randomized controlled trials, it was concluded that chocolate and cocoa appear to reduce both SBP and DBP after chronic intake [31]. In this regard, the results of this study are in line with previous evidence.

The decrease in SBP and PP observed in the participants with overweight or obesity of the IG with respect to those of the CG is in contrast to the results of another study, in which it was observed that the acute consumption of cocoa increased 4 mm Hg the BP at rest in healthy overweight subjects [18]. However, we must take into account that, in our study, the acute effects were not evaluated, but those in the medium term, and that the study population only included postmenopausal women.

Despite the fact that no significant differences were found, the results suggest that the intake of chocolate on its own or mixed with other foods or drinks could influence the possible effects of this compound, in the sense that the intake of other foods along with chocolate could interfere in the possible effects of chocolate on health. Nevertheless, it would be necessary to carry out other studies that could provide evidence on this.

The sample size of this trial was estimated based on the potential modification of the SBP, but it was not addressed to detect differences in the subgroup analysis. Therefore, it is possible that the sizes of the subgroups based on the presence or absence of overweight or obesity as baseline condition and by the mode of chocolate intake were inadequate for such analysis.

### 4.3. Discussion of the Results of Other Cardiovascular Risk Factors

With respect to metabolic risk factors, no changes were observed in the lipid profile in a similar way as in other studies with postmenopausal women [16], although a study with hypertensive patients with impaired glucose tolerance showed a significant decrease in the levels of TC and LDL cholesterol after the consumption of dark chocolate [32]. Furthermore, Okamoto et al. observed a significant decrease in triglycerides and glucose after the intake of cocoa [16], whereas our results showed no changes in these parameters. Previous studies indicate an improvement in the levels of insulin and HOMA-IR [15,32]; however, the results of the present study showed no differences in these parameters in any of the two groups or between them. Body weight remained unaltered after the consumption of dark chocolate, as shown by other studies performed in populations with augmented risk [16,18,33].

### 4.4. Discussion of the Results of Arterial Stiffness Parameters and Vascular Function

There is evidence that supports the improvement of arterial stiffness through the decrease of PWV after the consumption of cocoa, both in healthy subjects [19,30] and in postmenopausal women [16]. However, the results of the present study did not show significant changes.

Previous studies suggest that the consumption of chocolate with a high concentration of cocoa may improve vascular function in postmenopausal women [17], although Marsh et al. evaluated the acute effects, whereas the present study analyzed the effects in the medium term. Furthermore, the beneficial effects of a greater consumption of chocolate on the increase rate have been observed in healthy subjects [30] and in overweight women [18]. On the other hand, the parameters of vascular function measured in our study did not show relevant changes.

Our study has a larger sample of postmenopausal women compared to other similar studies with a relatively small sample size [16,17]. Moreover, the follow-up time of six months, which is in contrast to that of other studies in which the intervention lasted a few weeks, allowed us to evaluate the effects in the medium term.

The adherence to chocolate consumption reached in our study was practically total or very high, which is in line with that reported by other studies of similar characteristics [19,34,35,36].

With respect to the product used in the intervention, it is a commercially available compound with certain unmodifiable characteristics. Moreover, the contribution of 10 g of chocolate suits the recommendations of the European Food Safety Authority, which states that the consumption of this amount of high-flavanol dark chocolate included in a balanced diet could help maintain endothelium-dependent vasodilation [37]. However, other studies used a greater amount of chocolate, e.g., 50–100 g/day [31], or a product specifically manufactured for the purpose of the research, creating compounds with a very high content of polyphenols [17,19]. In this way, the possible effects can be potentiated with the great contribution of polyphenols, although this type of intervention does not fit a real clinical and easily reproducible context.

Likewise, there were no modifications or restrictions in the habitual diet of the participants in the present study, unlike in other studies [16]; the chocolate was simply added to the diet of the IG’s participants. This, along with the fact that the chocolate used is commercially available, allowed evaluating an intervention that could be applied in the usual clinical practice with a product that is accessible to the general population.

### 4.5. Limitations

The present study has several limitations. First, the amount of chocolate administered to the IG contained a low concentration of polyphenols compared to other studies. The polyphenol contribution of this amount of chocolate could be insufficient to show relevant changes in the effect size.

Moreover, it would have been interesting to evaluate the bioavailability of polyphenols by measuring, for example, epicatechin in plasma, with the aim of establishing a correlation with the results; however, this was not feasible.

The blinding of the participants was not possible due to the nature of the intervention, although the researchers who made the measurements and those who conducted the statistical analyses were blinded.

## 5. Conclusions

The daily intake of 10 g of cocoa-rich chocolate seems to provide little improvement to cardiovascular health, but neither does it cause any adverse effects on the parameters evaluated in postmenopausal women in the long term. However, it is necessary to carry out further clinical trials that include a greater number of subjects, easily reproducible in the usual clinical practice, and which evaluate the effects of the intake of cocoa-rich chocolate in the long term in populations with augmented cardiovascular risk, such as postmenopausal women.

## Figures and Tables

**Table 1:** Polyphenol composition of 10 g of 99% cocoa chocolate used in the intervention.

Compounds	Quantity, mg/10 g
Protocatechuic acid	0.58
Procyanidin dimer (B3)	1.76
Catechin	10.4
Procyanidin dimer (B2)	14.4
Epicatechin	26.1
Procyanidin trimer (C1)	8.53
Procyanidin A hexoside	3.54
Quercetin glucoside	0.02
Quercetin arabinoside	0.03

**Table 2:** Baseline characteristics of the postmenopausal women included in the study ^1^.

Variables	Intervention Group (*n* = 73)	Control Group (*n* = 67)
Age, y	57.1 ± 3.5	57.5 ± 3.8
Civil status, *n* (%)		
Married/cohabitant	48 (65.8)	47 (70.1)
Separated/divorced	8 (11.0)	7 (10.4)
Single	15 (20.5)	9 (13.4)
Widow	2 (2.7)	4 (6.0)
Education level, *n* (%)		
Elementary education	16 (21.9)	12 (17.9)
Middle-High school	22 (30.1)	29 (43.3)
Bachelor	17 (23.3)	11 (16.4)
Postgraduate	18 (24.7)	15 (22.4)
Time from menopause onset, y	6.9 ± 4.6	6.9 ± 3.6
Untreated hypertension, *n* (%)	1 (1.4)	0 (0.0)
Untreated dyslipidemia, *n* (%)	8 (11.0)	10 (14.9)
Gestational diabetes, *n* (%)	3 (4.1)	1 (1.5)
Thyroid hormone treatment, *n* (%)	13 (17.8)	10 (14.9)
Current smoker, *n* (%)	12 (16.4)	9 (13.4)
Alcohol consumption, g/week	23.1 ± 29.4	30.6 ± 48.1
Energy, kcal/day	1720 ± 357	1780 ± 402
Carbohydrates, g/day	168 ± 45.1	173 ± 50.2
Proteins, g/day	76.7 ± 16.7	78.2 ± 18.9
Fiber, g/day	24.0 ± 7.5	25.9 ± 9.6
Fats, g/day	77.4 ± 20.7	79.8 ± 20.1
Saturated fats, g/day	25.1 ± 7.6	25.3 ± 7.5
Physical activity, MET–h/week	31.2 ± 36.8	25.7 ± 20.0
Chocolate intake, g/week	68.6 ± 71.1	69.1 ± 74.2
>70% cocoa chocolate intake, g/week	19.9 ± 36.2	15.6 ± 33.2

^1^ Values are means ± SDs or frequencies (percent). MET, metabolic equivalent of task.

**Table 3:** Cardiovascular risk factors and blood pressure in postmenopausal women participants ^1^.

	Intervention Group (*n* = 71)				Control Group (*n* = 66)							
Characteristic	Baseline	6 Months	Change	*p* ^2^	Baseline	6 Months	Change	*p* ^2^	Intergroup Difference (IG-CG) ^3^	*p* ^3^	Adjusted Intergroup Difference (IG-CG) ^4^	*p* ^4^
Body weight, kg	65.1 ± 10.3	64.9 ± 10.3	−0.2 ± 2.2	0.438	64.9 ± 8.6	64.5 ± 8.9	−0.4 ± 2.7	0.272	0.16 (−0.67, 0.98)	0.708	0.16 (−0.66, 0.99)	0.696
BMI, kg/m^2^	25.7 ± 3.8	25.6 ± 3.7	−0.1 ± 0.9	0.502	25.6 ± 3.1	25.4 ± 3.2	−0.1 ± 1.0	0.250	0.07 (−0.25, 0.40)	0.652	0.08 (−0.24, 0.40)	0.627
Glucose, mg/dL	86.4 ± 8.8	86.6 ± 10.0	0.2 ± 7.9	0.820	86.2 ± 8.5	87.2 ± 8.7	1.0 ± 7.4	0.270	−0.80 (−3.39, 1.79)	0.542	−0.74 (−3.19, 1.70)	0.549
Insulin, mg/dL	8.2 ± 3.4	8.3 ± 5.1	0.1 ± 5.1	0.869	7.5 ± 2.9	7.8 ± 3.6	0.3 ± 3.0	0.434	−0.19 (−1.63, 1.24)	0.791	0.11 (−1.27, 1.48)	0.879
TC, mg/dL	211 ± 28.5	212 ± 34.6	1.3 ± 23.4	0.635	204 ± 26.6	205 ± 30.2	0.9 ± 18.5	0.700	0.44 (−6.71, 7.60)	0.902	1.12 (−6.06, 8.30)	0.758
HDL cholesterol, mg/dL	68.2 ± 17.3	67.0 ± 15.9	−1.2 ± 14.0	0.479	65.8 ± 13.2	65.0 ± 12.9	−0.9 ± 7.5	0.356	−0.32 (−4.16, 3.52)	0.870	0.45 (−3.00, 3.91)	0.795
LDL cholesterol, mg/dL	128 ± 26.4	130 ± 29.1	2.7 ± 16.9	0.175	122 ± 26.9	124 ± 29.3	1.6 ± 17.7	0.468	1.15 (−4.69, 7.00)	0.696	1.73 (−4.07, 7.54)	0.556
Triglycerides, mg/dL	83.4 ± 30.6	83.1 ± 34.7	−0.3 ± 27.4	0.938	80.0 ± 34.3	80.3 ± 28.5	0.2 ± 24.1	0.935	−0.49 (−9.24, 8.22)	0.911	0.63 (−7.35, 8.60)	0.876
HOMA-IR	1.8 ± 0.8	1.8 ± 1.4	0.1 ± 1.4	0.676	1.6 ± 0.7	1.7 ± 0.9	0.1 ± 0.7	0.292	−0.02 (−0.42, 0.36)	0.896	0.03 (−0.35, 0.41)	0.874
Creatinine, mg/L	0.7 ± 0.1	0.7 ± 0.1	0.0 ± 0.1	0.918	0.7 ± 0.1	0.7 ± 0.1	0.0 ± 0.1	0.242	0.02 (−0.02, 0.05)	0.336	0.03 (0.00, 0.05)	0.084
SBP, mm Hg	108 ± 16.4	106 ± 14.1	−1.8 ± 10.2	0.152	108 ± 15.0	108 ± 14.4	−0.2 ± 12.0	0.919	−1.62 (−5.39, 2.16)	0.398	−1.45 (−4.79, 1.88)	0.391
DBP, mm Hg	72.6 ± 10.7	72.4 ± 10.0	−0.3 ± 7.3	0.757	72.2 ± 10.3	71.4 ± 10.1	−0.7 ± 7.9	0.463	0.45 (−2.13, 3.03)	0.732	0.59 (−1.76, 2.94)	0.622
HR, bpm	66.5 ± 7.6	66.3 ± 8.0	−0.2 ± 6.0	0.804	66.7 ± 8.6	65.7 ± 7.6	−1.0 ± 7.6	0.298	0.80 (−1.51, 3.11)	0.495	0.74 (−1.31, 2.79)	0.479
PP, mm Hg	35.6 ± 9.5	34.1 ± 7.4	−1.5 ± 7.2	0.088	35.6 ± 7.6	36.1 ± 7.7	0.6 ± 7.1	0.518	−2.07 (−4.50, 0.37)	0.095	−2.05 (−4.08, −0.02)	0.048
MAP, mm Hg	84.5 ± 12.1	83.7 ± 11.0	−0.8 ± 7.7	0.403	84.0 ± 11.5	83.5 ± 11.2	−0.5 ± 8.9	0.629	−0.24 (−3.05, 2.56)	0.865	−0.10 (−2.63, 2.44)	0.939

^1^ Values are means ± SDs and differences are means (95% CI). BMI, body mass index; bpm, beats per minute; DBP, diastolic blood pressure; HDL, high-density lipoproteins; HOMA-IR, homeostasis assessment model for insulin resistance; HR, heart rate; LDL, low-density lipoproteins; MAP, mean arterial pressure; PP, pulse pressure; SBP, systolic blood pressure; TC, total cholesterol. ^2^ Intragroup comparison by the paired Student’s *t*-test. ^3^ These values are unadjusted. Intergroup comparison by the Student’s *t*-test. ^4^ These values are adjusted for baseline values. Results are based on ANCOVA.

**Table 4:** Arterial stiffness parameters and vascular function in postmenopausal women participants ^1^.

	Intervention Group (*n* = 71)				Control Group (*n* = 66)							
Characteristic	Baseline	6 Months	Change	*p* ^2^	Baseline	6 Months	Change	*p* ^2^	Intergroup Difference (IG-CG) ^3^	*p* ^3^	Adjusted Intergroup Difference (IG-CG) ^4^	*p* ^4^
CAVI	7.55 ± 0.91	7.78 ± 0.86	0.23 ± 0.67	0.005	7.70 ± 0.83	7.74 ± 0.87	0.04 ± 0.63	0.580	0.18 (−0.03, 0.40)	0.100	0.14 (−0.06, 0.34)	0.175
ABI	1.09 ± 0.07	1.11 ± 0.07	0.02 ± 0.07	0.064	1.10 ± 0.07	1.10 ± 0.07	0.01 ± 0.08	0.523	0.01 (−0.02, 0.03)	0.477	0.01 (−0.01, 0.03)	0.480
baPWV, m/s	12.1 ± 1.53	12.3 ± 1.55	0.14 ± 0.92	0.220	12.3 ± 1.53	12.3 ± 1.68	-0.08 ± 1.01	0.538	0.21 (−0.11, 0.54)	0.200	0.18 (−0.14, 0.50)	0.263
CAIx	41.6 ± 20.5	47.3 ± 29.1	5.77 ± 35.06	0.196	43.2 ± 19.4	47.9 ± 18.9	4.79 ± 22.3	0.092	0.98 (−9.38, 11.3)	0.852	−0.34 (−8.96, 8.27)	0.937
CAIx75	31.2 ± 15.4	35.5 ± 21.8	4.33 ± 26.3	0.196	32.4 ± 14.6	36.0 ± 14.2	3.59 ± 16.7	0.092	0.73 (−7.03, 8.50)	0.852	−0.26 (−6.72, 6.20)	0.937
PAIx	100.8 ± 16.1	103.5 ± 19.1	2.70 ± 26.0	0.407	101.6 ± 16.2	102.6 ± 18.0	0.97 ± 22.9	0.740	1.73 (−6.91, 10.4)	0.693	0.87 (−5.69, 7.42)	0.794

^1^ Values are means ± SDs and differences are means (95% CI). ABI: ankle-brachial index; ba-PWV: brachial-ankle pulse wave velocity; cAIx: central augmentation index; cAIx75: central augmentation index corrected for a heart rate of 75 bpm; CAVI: cardio-ankle vascular index; pAIx: peripheral augmentation index. ^2^ Intragroup comparison by the paired Student’s *t*-test. ^3^ These values are unadjusted. Intergroup comparison by the Student’s *t*-test. ^4^ These values are adjusted for baseline values. Results are based on ANCOVA.
